# Targeted review of *IL36RN* mutations in patients with generalised pustular psoriasis

**DOI:** 10.1002/ski2.343

**Published:** 2024-03-06

**Authors:** James G. Krueger, Anna Pagotto, Samuel Haftel, Birgit Gradl

**Affiliations:** ^1^ Laboratory for Investigative Dermatology The Rockefeller University New York New York USA; ^2^ Symmetron Limited London UK; ^3^ Boehringer Ingelheim International GmbH Ingelheim am Rhein Germany

## Abstract

**Background:**

Generalised pustular psoriasis (GPP) is a rare and chronic skin disease historically treated with therapies that were originally intended to treat plaque psoriasis (PsO). However, GPP and plaque PsO have distinct pathogeneses and clinical courses.

**Objectives:**

This study aimed to further characterise the unique genetic background of GPP by summarising evidence on the frequency and type of *IL3*6RN gene mutation, a gene that normally suppresses proinflammatory responses, in patients with GPP compared to patients with GPP and plaque PsO, and patients with plaque PsO only.

**Methods and Results:**

A targeted literature review was conducted to identify studies reporting *IL3*6RN mutations and/or *HLA‐Cw6* allele frequency in patients with GPP. Meta‐analyses showed a significantly higher rate of *IL3*6RN mutations in the GPP‐only population compared to the GPP + plaque PsO population (OR 3.51; 95% CI 2.29, 5.38). Monoallelic mutations of *IL3*6RN were found in up to 33.3%, and biallelic mutations in up to 73.2% of patients with GPP (GPP‐only and GPP + plaque PsO), in contrast with mono‐ and biallelic frequencies of only 0%–11.9% and 0%, respectively, in patients with plaque PsO only. Mean age‐of‐onset ranged from 5.9 to 48.9 years old, with most studies reporting a GPP age‐of‐onset between 20 and 40 years old. Twenty‐one mutations were identified in the biallelic state and three in monoallelic. The most reported mutations were c.115 + 6T > C (p. Arg10ArgfsX1) (18 studies); c.227 C > T (p.Pro76Leu) (10 studies); and c.338 C > T (p.Ser113Leu) (8 studies). Mutations varied depending on geography and ethnicity, with the most frequently reported mutation predominantly reported in East Asian studies and international studies that included Asian patients. Rates of *HLA‐Cw6*, the risk allele most strongly associated with plaque PsO, were 0%–28.6% for patients with GPP, similar to rates in the general population (10.5%–20%).

**Conclusion:**

Considering the differences between GPP and plaque PsO in aetiology and disease symptoms, effective, GPP‐specific treatment options are needed, and recent research suggests that blockade of IL‐36 signalling may be an effective target for treatment of GPP.

## Graphical abstract

Generalised pustular psoriasis (GPP) is a rare, chronic, skin disease often treated with therapies used to treat plaque psoriasis (PsO). However, GPP and plaque PsO have distinct pathogeneses and clinical courses. This study summarises evidence on the frequency and types of *IL36RN* gene mutation in patients presenting only with GPP (GPP only) compared to patients with GPP and plaque PsO, and patients with plaque PsO only. In the targeted literature review, monoallelic and biallelic *IL36RN* mutations, respectively, were reported in up to 33.3% and 73.2% of patients with GPP and 0%–11.9% and 0% of patients with plaque PsO. Mutations varied depending on geography and ethnicity. Considering the unique pathogenesis of GPP, targeted and effective GPP‐specific treatment options are needed.

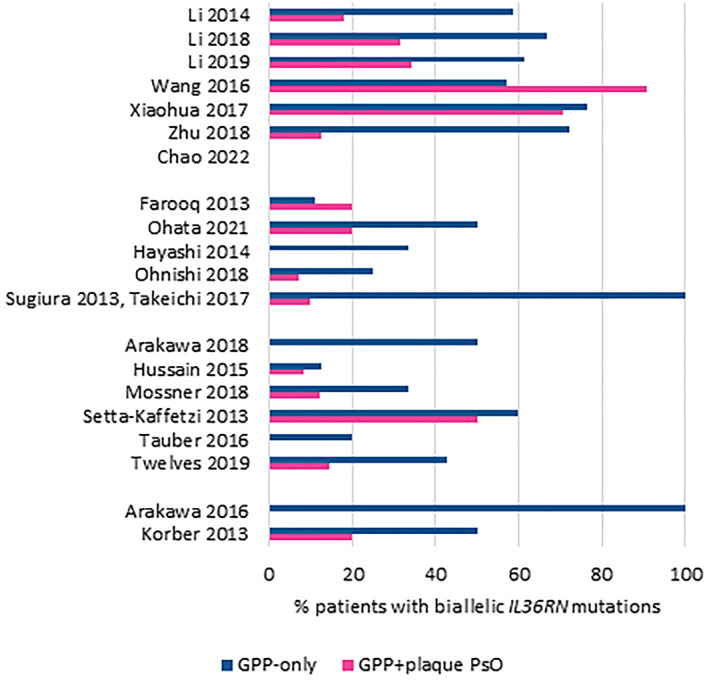

**What's already known about this topic?**

- Generalised pustular psoriasis (GPP) and plaque psoriasis (PsO) have distinct clinical characteristics and pathogeneses, and evidence suggests that their aetiologies may also be different. While the presence of *HLA‐Cw6* alleles have been associated with plaque PsO, mutations in *IL3*6RN and overactive IL36 signalling have both been observed in GPP.

**What does this study add?**

- This review and meta‐analysis found that rates of *IL3*6RN mutations were significantly higher in patients with GPP only versus patients with both GPP and plaque PsO or plaque PsO only. Moreover, biallelic mutations *IL3*6RN were more common than monoallelic mutations. In contrast, rates of *HLA‐Cw6* alleles linked to plaque PsO were similar between the GPP population and the general population. Our study suggests that the genetic aetiology of GPP differs from plaque PsO.

## 1 INTRODUCTION

Generalised pustular psoriasis (GPP) is a rare, chronic, inflammatory skin disease with recurring flares of sterile pustules and widespread erythema.^1^, ^2^, ^3^ Flares can result in potentially life‐threatening complications, including sepsis or heart failure.^4^, ^5^ The prevalence of GPP is not well characterised, but estimates range from 1.76 per million to 124 per million globally.^2^, ^5^ One of the most common genetic mutations correlated with the development of GPP occurs in the *IL3*6RN gene that encodes the interleukin‐36 receptor antagonist (IL‐36Ra) protein, which normally suppresses proinflammatory responses.^1^, ^2^, ^6^ The IL‐36 pathway is regulated through the dynamic interaction between IL‐36 agonists and antagonists, ensuring a harmonised immune response. Dysregulation, such as the overexpression of IL‐36 or impaired functionality of IL‐36Ra, leads to an aberrant inflammatory response.^7^, ^8^ The hallmark pustules of GPP result from overactivation of the IL‐36 pathway leading to the secretion of inflammatory mediators that promote skin infiltration of neutrophils and ultimately the development of incredibly painful, sterile, pus‐filled pustules that may take weeks to subside.^2^, ^3^, ^5^, ^6^, ^9^

GPP, International Classification of Diseases (ICD) code L40.1, can occur with or without concomitant plaque psoriasis (i.e., plaque PsO, ICD L40.0), the most common form of PsO. Historically, GPP has been diagnosed and treated similarly to plaque PsO despite its different presentation and clinical course. Recently, researchers have acknowledged the distinct pathogenesis of GPP and need for differential diagnoses and treatments.^2^, ^9^ Studies have suggested that the presence of *HLA‐Cw6* alleles is a significant genetic factor that has been strongly associated with susceptibility to both plaque PsO and guttate PsO, but such an association has not been established for GPP.^10^, ^11^, ^12^ *HLA‐Cw6* has been observed to affect age‐of‐onset, disease course, phenotype, severity, comorbidities, and treatment outcomes for PsO.^10^

Immunomodulatory therapies, including biologics, are used mostly to treat GPP based mainly on clinical experience in patients with plaque PsO; however, evidence regarding their efficacy and safety for the treatment of GPP is limited.^4^, ^5^ Recent research has demonstrated that blockade of IL‐36 signalling is an effective target for treatment of GPP.^3^, ^6^ In the pivotal Effisayil^TM^ 1 trial, the first and largest placebo‐controlled study of 53 patients with GPP, treatment with the IL‐36‐receptor inhibitor spesolimab was associated with complete pustule clearance (Generalised Pustular Psoriasis Physician Global Assessment [GPPGA] subscore of 0) after 1 week of treatment (19 of 35 patients [54%] in the spesolimab arm compared with 1 of 18 patients in the placebo arm [6%]).^13^

The objective of this study was to highlight differences in the genetic background of GPP compared to plaque PsO by identifying and summarising evidence on the frequency and type of *IL3*6RN mutations and *HLA‐Cw6* allele prevalence in patients with GPP. To further characterise the unique genetic background of GPP, *IL3*6RN mutation rates for patients with GPP only were compared to those with plaque PsO only and those with GPP and concomitant plaque PsO (GPP + plaque PsO). By examining these genetic factors, we aimed to better understand the distinct pathogenic mechanisms and contributing factors that differentiate these two forms of PsO.

## 2 METHODS

A targeted literature review was conducted on 8 April 2021 and updated on 28 September 2021 and 4 August 2022 to identify all studies published until that date reporting evidence on the frequency and type of *IL3*6RN mutations and *HLA‐Cw6* allele prevalence in GPP patients. Records were accessed via Excerpta Medica database (Embase), Medical Literature Analysis and Retrieval System Online (Medline), Medline In‐Process, and Cochrane Library (see Tables S1‐S6 for search strategies). Conference searches included National Organisation for Rare Diseases (NORD), European Conference on Rare Diseases (ECRD), International Congress of Research on Rare and Orphan Diseases (RE[ACT]), and European Academy of Dermatology and Venereology (EADV). American Academy of Dermatology (AAD) was not searched separately as it is captured in Embase. Titles and abstracts were assessed for inclusion against pre‐defined population, intervention, comparators, outcomes, study types (PICOS) criteria and relevant full texts were further examined against these eligibility criteria (Table 1). Full texts were independently screened by two reviewers and discrepancies resolved through discussion. For excluded studies, the primary reason for exclusion was documented (Table S7). Study characteristics, patient characteristics (including associated plaque PsO and GPP age‐of‐onset), *IL3*6RN mutations (rates and types), and *HLA‐Cw6* allele prevalence were extracted and analysed.

**TABLE 1:** Population, intervention, comparators, outcomes, study types (PICOS) criteria.

PICOS	Criteria for inclusion	Criteria for exclusion
Population	Patients described as having any of the following: GPP (general GPP or any GPP subtype defined according to any criteria) Acute GPP (Von Zumbusch) GPP flare Acute phase of GPP flare Infantile/juvenile pustular PsO	Subsets of PsO patients with: Non‐pustular PsO Localised pustular PsO (including palmoplantar pustular PsO) Synovitis‐acne‐pustulosis‐hyperostosis‐osteitis syndrome Erythrodermic plaque PsO without pustules or with pustules restricted to psoriatic plaques Drug‐triggered acute generalised exanthematous pustulosis Impetigo herpetiformis (IH) Annular or circinate pustular PsO Subcorneal pustular dermatosis (SCPD, Sneddon‐Wilkinson disease)
Intervention	Any	None
Comparators	Any	None
Outcomes	Publications that report IL36RN mutations and/or *HLA‐Cw6* allele prevalence in GPP patients^a^	All other studies
Study types	Clinical trials Registry/database analyses Observational studies Literature reviews (non‐narrative)^b^ Consensus studies^b^	Case studies Case reports Narrative reviews Editorials

Abbreviations: GPP, generalised pustular psoriasis; PsO, psoriasis; PICOS, population, intervention, comparators, outcomes, study types.

^a^ In order to efficiently review evidence, reporting of mutations was assessed at the title/abstract stage. Articles that did not mention *IL3*6RN mutations or *HLA‐Cw6* prevalence in the title/abstract were excluded from the review. It is therefore possible that some publications that mentioned these outcomes in their full text, but not in their abstract or title, may have been missed from this review.

^b^ Bibliographies of included literature reviews and consensus studies were reviewed to determine whether they contained any relevant studies that met the inclusion criteria. The results and data used in the literature reviews and consensus studies themselves were not of interest.

### 2.1 Statistical analyses

Four analyses were conducted to explore and compare rates of *IL3*6RN mutations. Two analyses assessed *IL3*6RN mutation rates, and two analyses explored GPP age‐of‐onset. The first set of analyses compared rates of any *IL3*6RN mutations (i.e., monoallelic and biallelic pooled) in patients with GPP only versus patients with GPP + plaque PsO, while the second set of analyses compared rates of biallelic mutations‐only across these two populations. The third and fourth sets of analyses compared the age of disease onset in patients with or without biallelic mutations in the overall GPP population, and in the GPP‐only subgroup versus the GPP + plaque PsO subgroup. Under each analysis, one meta‐analysis comparing subgroups and one meta‐analysis of overall population were performed.

As each analysis contained ≥10 studies, publication bias was assessed using funnel plots and Egger's test for meta‐analyses comparing subgroups. When >1 study from the same research group was identified, only the most recent study was included in the meta‐analyses to prevent unintentional bias introduced by overlapping patient data across studies. Studies were only included if they reported data (or data could be inferred) for the subgroups of interest. Additionally, studies that selected patients that were only from a certain population age group (e.g., paediatric or elderly) were excluded, as were studies with <2 reported cases of age‐of‐onset, and familial‐only studies. In the meta‐analyses, studies were pooled via the Mantel‐Haenszel method. Heterogeneity was assessed using the *I* ^2^ statistic.

Regarding missing data, mean age of GPP onset and/or standard deviation were not reported and could not be calculated based on individual patient data for several studies.

Meta‐analyses that compared patient subgroups were conducted in RStudio (version 4.2.1)^14^ using the ‘meta’ package (version 5.5–0).^15^ Both fixed and random‐effects models were used; however, due to study heterogeneity in terms of country, study design, and number of included patients, the random‐effects model results were considered more appropriate estimates in this analysis.

Meta‐analyses of proportions were conducted for *IL3*6RN mutations and biallelic *IL3*6RN mutations in RStudio using the ‘metaprop’ function (meta package). Data were transformed using a logit function and a random‐intercept logistic regression model was used to conduct the meta‐analysis.

Meta‐analyses of single means were conducted for age of disease onset in RStudio using the ‘metamean’ function (meta package). Fixed and random‐effects models were explored, and inverse‐variance weighting was used to pool studies.

## 3 RESULTS

**FIGURE 1:**
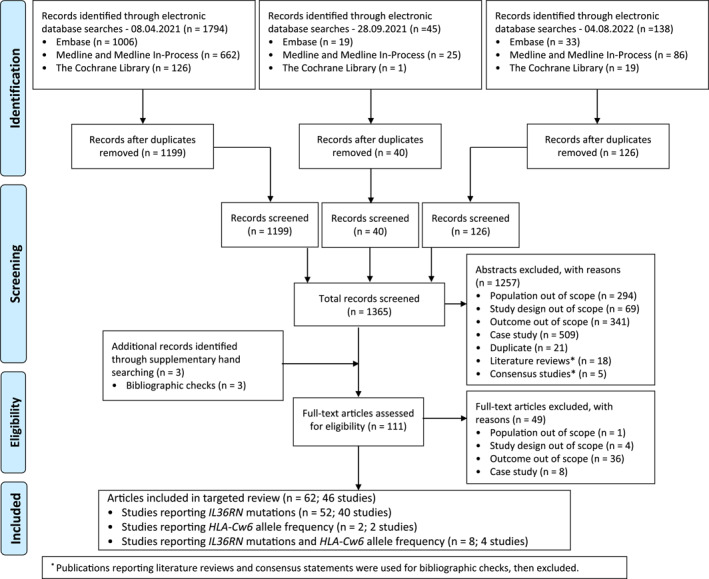
PRISMA flow diagram. PRISMA, Preferred Reporting Items for Systematic Reviews and Meta‐Analyses.

Of a total of 1977 initial records identified, 46 studies were included (Figure 1). Among them, 40 studies reported *IL3*6RN mutations only, two studies reported *HLA‐Cw6* allele only, and four studies reported both.

About 22 studies included were conducted in East Asia (China, Japan, Malaysia), and 13 were international studies conducted in multiple countries (Table 2). Study sample size varied between 4 and 210 patients.

**TABLE 2:** Overview of included studies.

Study ID	Country	Study design	GPP patients (*N*)	Type of GPP	Mean age—years (SD)^a^	Sex ‐ M/F (ratio)^a^
China						
Li 2019	China	NR	107	NR	NR	NR
Li 2013a	China	Observational	68	NR	29.6 (22.1)	48/20 (2.4)
Wang 2017	China	Observational	66^b^	Sporadic	8.5 (4.3)	47/19 (2.5)
Li 2014	China	Observational	62	Sporadic	36.7 (17.5)	38/24 (1.6)
Zhu 2018	China	Observational	61	NR	NR	33/28 (1.2)
Liang 2017	China	Observational	56	NR	8.2 (4.5)	44/12 (3.7)
Li 2018	China	Observational	43	NR	35.2 (17.3)	25/18 (1.4)
Xiaohua 2017	China	NR	41	NR	NR	NR
Wang 2016	China	Observational	32	NR	32.9 (14.9)	12/20 (0.6)
Li 2013b	China	Observational	10	Sporadic	NR	6/4 (1.5)
Chao 2022	China	Observational	7^c^	Sporadic	74.3 (7.6)	3/4 (0.75)
Japan						
Ohata 2021	Japan	Observational	102	NR	58.0 (17.8)	53/49 (1.1)
Sugiura 2013, Takeichi 2017	Japan	Observational	31	Sporadic and familial	NR	18/13 (1.4)
Manone‐Zenke 2022	Japan	Observational	27	NR	NR	11/16 (0.7)
Ozawa 1998	Japan	Observational	26	Sporadic	NR	NR
Okubo 2018	Japan	Observational	25	NR	54.0 (NR)	14/15 (0.9)
Ohnishi 2018	Japan	Observational	22	NR	53.4 (14.0)	9/13 (0.7)
Farooq 2013	Japan	Observational	14	Sporadic	52.6 (21.6)	9/5 (1.8)
Uchida 2022	Japan	Observational	14	NR	64.5 (12.6)	8/6 (1.3)
Muto 2014	Japan	Observational	10^d^	Sporadic	NR	NR
Hayashi 2014	Japan	Observational	8	Sporadic	50.1 (16.0)	2/6 (0.3)
International						
Twelves 2017	International (authors are from UK, Hungary, Malaysia, Ireland, Singapore)	Observational	210	NR	NR	NR
Twelves 2019	International (8 patient cohorts: UK/Ireland; Malaysia; Austria; Egypt; Switzerland; Germany; Hungary; Estonia; others)	Observational	191	Sporadic	NR	59/128 (0.5)^e^
Madrange 2014	International (authors are from France, Italy, Spain)	Observational	100	NR	NR	NR
Setta‐Kaffetzi 2013	International (patients of European and Asian origin)	Observational	84	Sporadic	NR	29/55 (0.5)
Mossner 2018	International (Europe, Turkey, Syria, Morocco, Palestine, a foreign country not further specified, and two patients had grandparents from Iraq)	Observational	61	Sporadic and familial	47.5 (17.5)	18/43 (0.4)
Hussain 2015	International (UK/Ireland, China, India, Malaysia, Ethiopia, Poland, Bangladesh, Italy, Germany, Sri Lanka, Switzerland, Algeria, Spain, Tunisia, Korea)	Mixed analysis (SLR + patients diagnosed by co‐authors + patients ascertained by the international registry of severe cutaneous Adverse reaction consortium)	56	NR	NR	18/38 (0.5)
Twelves 2016	International (authors are from UK, Hungary, Malaysia, Germany)	Observational	43	Sporadic	NR	NR
Ammar 2016	International (Tunisia and Europe)	Observational	16^f^	Sporadic and familial	NR	NR
Tauber 2016	International (authors are from France, UK, Algeria, Tunisia)	Observational	14^g^	Sporadic and familial	NR	4/6 (0.7)^h^
Magalhaes 2022	International (authors are from Brazil and UK)	Observational	13	NR	NR	4/9 (0.4)
Arakawa 2018	International (Germany and Afghanistan)	Observational	8	NR	50.3 (15.6)	1/7 (0.1)
Arakawa 2013	International (authors are from Japan and Germany)	Observational	7	NR	NR	NR
Bachelez 2019	International (France, Japan, Korea, Malaysia, Taiwan, Tunisia)	Clinical trial (phase I)	7	NR	38.6 (13.8)	3/4 (0.7)
Germany						
Korber 2013	Germany	Observational	17	NR	46.6 (14.4)	5/12 (0.4)
Arakawa 2014	Authors are from Germany	Observational	8	NR	NR	NR
Wilsmann‐Theis 2018	Germany	Observational	7	NR	56.9 (13.9)	2/5 (0.4)
Arakawa 2016	Authors are from Germany	Observational	4	NR	NR^i^	0/4 (0.0)
Tunisia						
Gharbi 2019	Tunisia	Observational	44	Sporadic and familial	NR^j^	NR (0.9)
Marrakchi 2011	Tunisia	Observational	16	Familial	34.4 (15.2)	6/10 (0.6)
Bougacha 2016	Tunisia	Observational	NR^k^	Familial	NR	NR
Tauber 2014	Tunisia/Maghreb	Observational	NR^l^	Familial	NR	NR
Other						
Huffmeier 2015	NR	Observational	45	NR	NR	NR
Lau 2017	Malaysia	Observational	27^b^	NR	20.5 (12.0)	11/16 (0.7)
Cardili 2016	Brazil	Observational	14^m^	NR	NR	NR
Onoufriadis 2011	Authors are from the UK	Observational	5	Sporadic	NR	1/4 (0.2)

Abbreviations: GPP, generalised pustular psoriasis; M/F, male/female; NR, not reported; SD, standard deviation; SLR, systematic literature review; UK, United Kingdom.

^a^ Values were calculated based on the individual patient data reported in the studies.

^b^ Only paediatric patients were included in the study.

^c^ Only elderly‐onset patients were included in the study.

^d^ Ten unrelated individuals and one family were included.

^e^ The gender of four patients is unknown.

^f^ Sixteen European patients and 2% of 282 Tunisian patients. For the entire Tunisian cohort, the mean age was 41.9, there were 178 males and 104 females.

^g^ Ten sporadic patients and four familial patients were included.

^h^ Reported for sporadic patients only.

^i^ Two patients in their twenties and two patients in their fifties.

^j^ Age ranged between 19 months and 67 years.

^k^ Eight families with different forms of psoriasis were included; unclear how many had GPP.

^l^ Two families were included.

^m^ Including GPP patients and erythrodermic psoriasis patients.

The age of GPP patients at the time of the study ranged between 8.2 and 74.3 years old; two studies only included paediatric patients,^16^, ^17^ while one study included elderly‐onset GPP patients.^18^ Thirty‐two studies reported patients' sex and most were female patients. In 33 studies, the authors reported whether associated plaque PsO was present in GPP patients (including previous and/or concomitant plaque PsO), and the age of GPP onset was reported in 25 studies. Eight studies also included a plaque PsO‐only patients cohort and examined *IL3*6RN mutations in this subgroup.^19^, ^20^, ^21^, ^22^, ^23^, ^24^, ^25^, ^26^

### 3.1 IL36RN mutation rates

The background frequency of *IL3*6RN mutations in the general population was assessed using cohorts of healthy individuals from six studies (range: 50–1130 individuals).^19^, ^20^, ^21^, ^22^, ^24^, ^27^ Among healthy controls, 0%–10.7% carried *IL3*6RN mutations. In studies with plaque PsO‐only patient cohorts, rates of monoallelic *IL3*6RN mutations ranged from 0% to 11.9%, and no biallelic mutations were found.^20^, ^21^, ^22^, ^23^, ^24^, ^25^, ^26^

**FIGURE 2:**
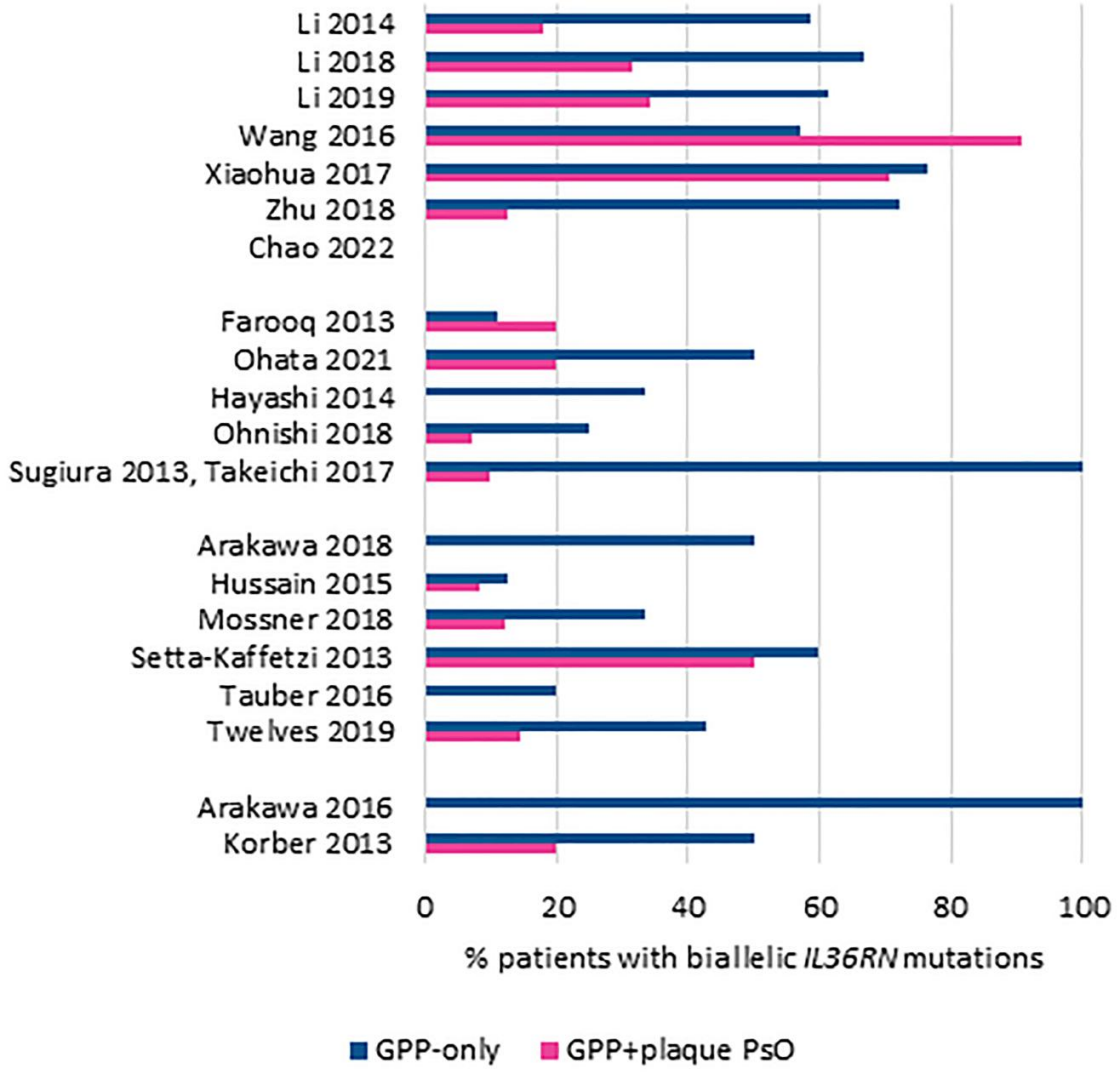
*IL3*6RN carrier rate of biallelic mutation in patients with GPP‐only versus GPP + plaque psoriasis. GPP, generalised pustular psoriasis.

**FIGURE 3:**
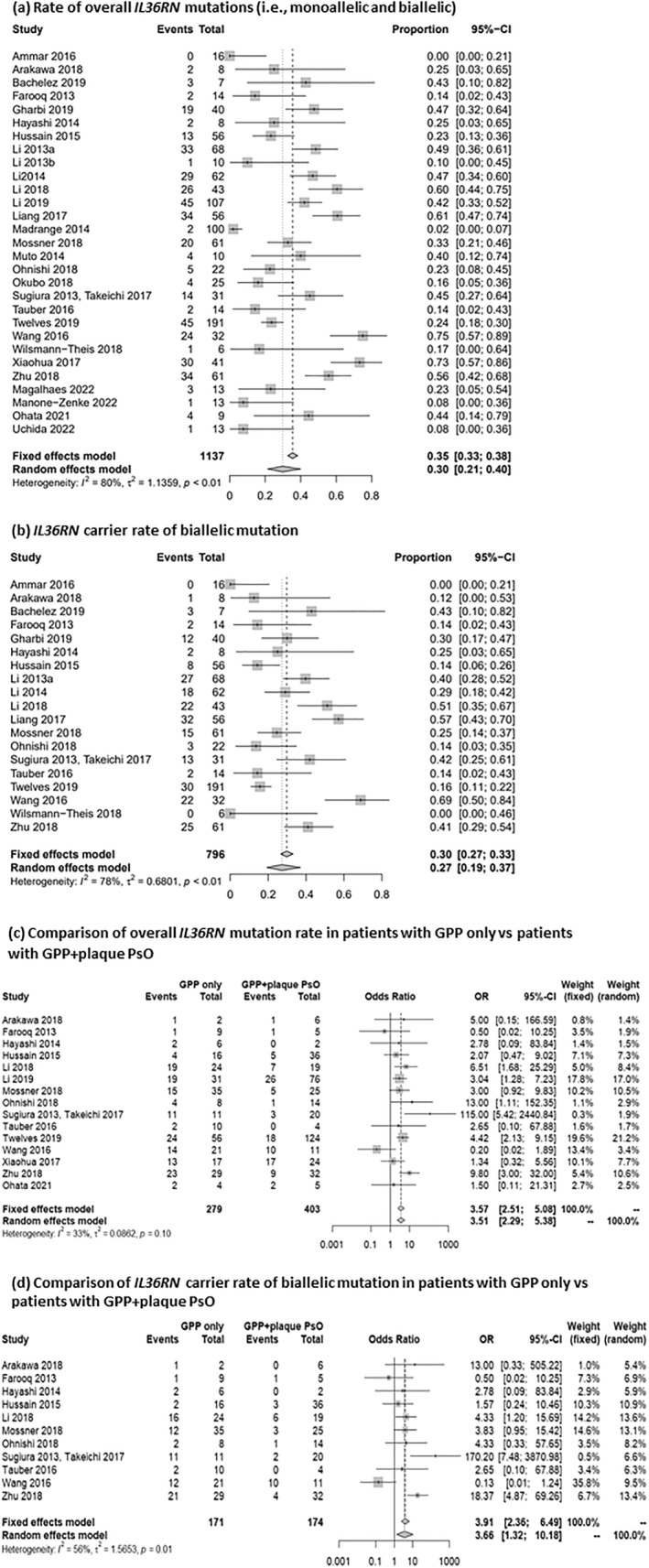
Meta‐analyses assessing *IL3*6RN mutations in patients with GPP. (a) Rate of overall *IL3*6RN mutations (ie, monoallelic and biallelic); (b) IL36RN carrier rate of biallelic mutation; (c) Comparison of overall *IL3*6RN mutation rate in patients with GPP only versus patients with GPP + plaque PsO; (d) Comparison of *IL3*6RN carrier rate of biallelic mutation in patients with GPP only versus patients with GPP + plaque PsO. CI, confidence interval; GPP, generalised pustular psoriasis; OR, odds ratio; PsO, psoriasis.

In contrast, biallelic mutations of *IL3*6RN were found in up to 73.2% of GPP patients and monoallelic mutations found in up to 33.3% of GPP patients (Table 3). Among 44 studies reporting *IL3*6RN mutations, 20 studies also reported the presence of associated plaque PsO. Seventeen of these 20 studies reported a higher percentage of biallelic mutations in the GPP‐only population than in patients with GPP + plaque PsO (Table 4, Figure 2). The likelihood of having *IL3*6RN mutations (either biallelic or monoallelic) was significantly higher for the GPP‐only population versus the GPP + plaque PsO population (OR 3.51; 95% CI 2.29, 5.38; Figure 3c). Similarly, the probability of having biallelic *IL3*6RN mutations was higher for the GPP‐only population versus the GPP + plaque PsO population (OR 3.66; 95% CI 1.32, 10.18; Figure 3d). Overall, approximately 30% of patients with GPP had *IL3*6RN mutations (95% CI 0.21, 0.40; Figure 3a), with an estimated 27% (95% CI 0.19, 0.37) of patients bearing biallelic mutations (Figure 3b). Chinese studies reported higher mutation rates.

**TABLE 3:** *IL3*6RN mutation rate overview.

Country	Number of studies	% GPP patients with *IL36RN* biallelic mutations^a^	% GPP patients with *IL36RN* monoallelic mutations^a^
China	11	0.0–73.2%^b^	3.6%–33.3%
Japan	9	7.7–41.9%^b^	0.0%–11.1%
International	13	0.0–42.9%^b^	0.0%–14.3%
Germany	4	0.0%–41.2%	0.0%–16.7%
Tunisia	4	30.0%^c^	17.5%
Other	3	29.0%–60.0%	0.0%–10.0%
**TOTAL**	**44**	**0.0%–73.2%**	**0.0%–33.3%**

*Note*: Total studies and percentage range are given in bold format.

Abbreviations: GPP, generalised pustular psoriasis.

^a^ Values were calculated based on the individual patient data reported in the studies.

^b^ Biallelic and monoallelic IL36RN mutations were reported pooled in three studies.

^c^ Bougacha 2016 and Marrakchi 2011 were excluded as they only included familial cases.

**TABLE 4:** *IL36RN* carrier rate of biallelic mutation and associated plaque PsO.

Study ID	GPP patients (*N*)	% GPP patients with associated plaque PsO (n/N)	%*IL36RN* biallelic mutations^a^	
			Among GPP‐only patients	Among GPP with plaque PsO patients
China				
Li 2019	107	71.0% (76/107)	61.3% (19/31)^b^ ^,^ ^c^	34.2% (26/76)^d^
Li 2014	62	72.6% (45/62)	58.8% (10/17)	17.8% (8/45)
Zhu 2018	61	52.5% (32/61)	72.4% (21/29)	12.5% (4/32)
Li 2018	43	44.2% (19/43)	66.7% (16/24)	31.6% (6/19)
Xiaohua 2017	41	58.5% (24/41)	76.5% (13/17)^b^ ^,^ ^c^	70.8% (17/24)^d^
Wang 2016	32	34.4% (11/32)	57.1% (12/21)	90.9% (10/11)
Chao 2022	6^e^	0% (0/6)	0% (0/6)	0% (0/0)
Japan				
Sugiura 2013, Takeichi 2017	31	64.5% (20/31)	100% (11/11)	10.0% (2/20)
Ohnishi 2018	22	63.6% (14/22)	25.0% (2/8)	7.1% (1/14)
Farooq 2013	14	35.7% (5/14)	11.1% (1/9)	20% (1/5)
Ohata 2021	9	55.6% (5/9)	50% (2/4)	20% (1/5)
Hayashi 2014	8	25% (2/8)	33.3% (2/6)	0.0% (0/2)
International				
Twelves 2019	191	65.4% (125/191)	42.9% (24/56)^c^ ^,^ ^f^	14.5% (18/124)^d^ ^,^ ^f^
Setta‐Kaffetzi 2013	84	61.5% (8/13)^g^	60.0% (3/5)	50.0% (4/8)
Mossner 2018	61	41.0% (25/60)^h^	34.3% (12/35)	12.0% (3/25)
Hussain 2015	56	69.2% (36/52)^i^	12.5% (2/16)	8.3% (3/36)
Tauber 2016	14	28.6% (4/14)	20.0% (2/10)	0.0% (0/4)
Arakawa 2018	8	75% (6/8)	50% (1/2)	0% (0/6)
Germany				
Korber 2013	17	29.4% (5/17)	50.0% (6/12)	20.0% (1/5)
Arakawa 2016	4	75% (3/4)	100.0% (1/1)	0.0% (0/3)

Abbreviations: GPP, generalised pustular psoriasis; PsO, psoriasis.

^a^ Values were calculated based on the individual patient data reported in the studies.

^b^ Biallelic and monoallelic mutations were only reported pooled.

^c^ Value corresponds to number of patients with at least 1 mutation without plaque PsO.

^d^ Value corresponds to number of patients with at least 1 mutation and plaque PsO.

^e^ Sequencing analysis was performed for six of the seven total patients; only the polymorphism c.115 + 6T > C was analysed because of its dominant role in Chinese patients with GPP.

^f^ This study only reported the plaque PsO status for 42 out of 45 patients of the pooled monoallelic and biallelic GPP.

^g^ Plaque PsO status was only reported for the 13 patients bearing *IL36RN* mutations.

^h^ Plaque PsO status was unclear for 1 patient.

^i^ Plaque PsO status unknown for 4 patients.

### 3.2 *IL36RN* mutation types

**FIGURE 4:**
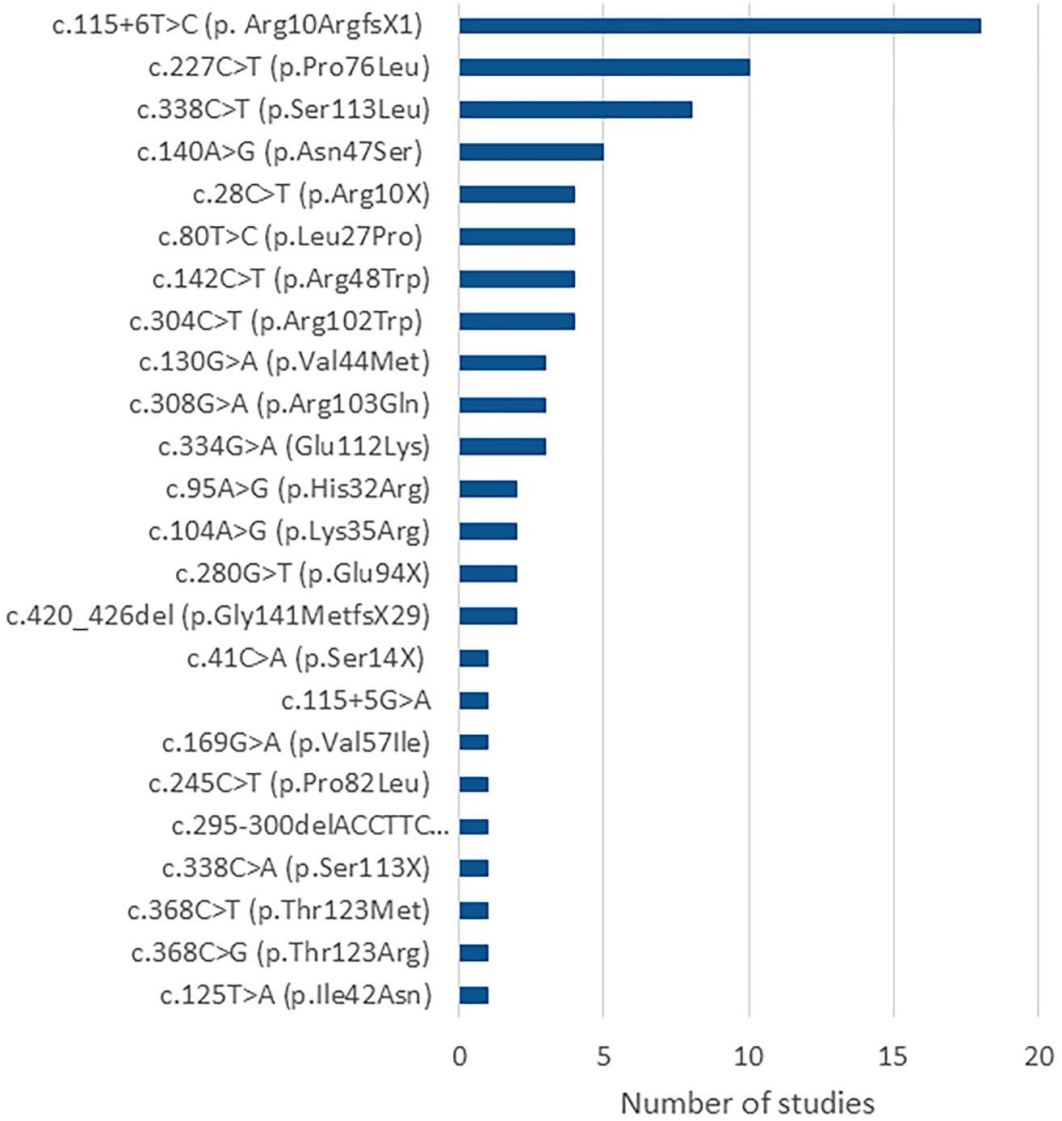
Number of studies reporting specific mutation types.

Twenty‐one mutations were identified in the biallelic state, including amino acid substitutions (13 mutations), protein truncations (4 mutations), splicing defects (2 mutations), and amino acid deletions (2 mutations) (Table 5). Six of these mutations were also found in monoallelic state. Three additional mutations were only found in the monoallelic state (3 substitutions) (Table 5). The most reported mutations were c.115 + 6T  >  C (p. Arg10ArgfsX1), reported in 18 studies; c.227 C > T (p.Pro76Leu), reported in 10 studies; and c.338 C > T (p.Ser113Leu), reported in 8 studies (Figure 4). When examining the patient populations reporting specific mutations, differences in ethnicity were noted. The c.115 + 6T > C (p. Arg10ArgfsX1) mutation was primarily reported in East Asian studies and international studies that included Asian patients (17/18 studies), and the c.338 C > T (p.Ser113Leu) mutation was mostly reported in European patients (7/8 studies). The c.28 C > T (p.Arg10X) mutation was exclusively reported in Japanese patients, and the c.80 T > C (p.Leu27Pro) mutation was exclusively reported in North‐African patients.

**TABLE 5:** *IL3*6RN mutation type and effect on the protein.

*IL36RN* mutations identified	Effect of mutation
c.115 + 6T > C (p. Arg10ArgfsX1)	Truncation
c.227 C > T (p.Pro76Leu)	Substitution
c.338 C > T (p.Ser113Leu)	Substitution
c.140 A > G (p.Asn47Ser)	Substitution
c.28 C > T (p.Arg10X)	Truncation
c.80 T > C (p.Leu27Pro)	Substitution
c.142 C > T (p.Arg48Trp)	Substitution
c.304 C > T (p.Arg102Trp)	Substitution
c.130 G > A (p.Val44Met)	Substitution
c.308 G > A (p.Arg103Gln)	Substitution
c.334 G > A (Glu112Lys)	Substitution
c.95 A > G (p.His32Arg)	Substitution
c.104 A > G (p.Lys35Arg)	Substitution
c.280 G > T (p.Glu94X)	Truncation
c.420_426del (p.Gly141MetfsX29)	Truncation
c.41 C > A (p.Ser14X)	Truncation
c.115 + 5G > A	Splicing defect
c.169 G > A (p.Val57Ile)	Substitution
c.245 C > T (p.Pro82Leu)	Substitution
c.295‐300delACCTTC (p.Thr99_Phe100del)	Deletion of 2 amino acids
c.338 C > A (p.Ser113X)	Truncation
c.368 C > T (p.Thr123Met)	Substitution
c.368 C > G (p.Thr123Arg)	Substitution
c.125 T > A (p.Ile42Asn)	Substitution

The c.115 + 6T > C (p. Arg10ArgfsX1) mutation, occurring within a splice site, has been shown to cause aberrant *IL3*6RN mRNA splicing resulting in the skipping of exon 3 leading to premature translation termination.^27^, ^28^, ^29^ The c.227 C > T (p.Pro76Leu) mutation is located at a loop position where a leucine could not be accommodated owing to steric clashes, causing structural changes of the respective loop and/or decreased protein stability; online prediction tools predicted this mutation to be damaging.^30^ The c.338 C > T (p.Ser113Leu) mutation affects an evolutionarily conserved IL36RN residue proximal to critical binding residues and is predicted by the Sorting Intolerant From Tolerant algorithm to be damaging, findings confirmed by in vitro assays.^23^, ^30^

### 3.3 GPP age‐of‐onset by mutational status

**FIGURE 5:**
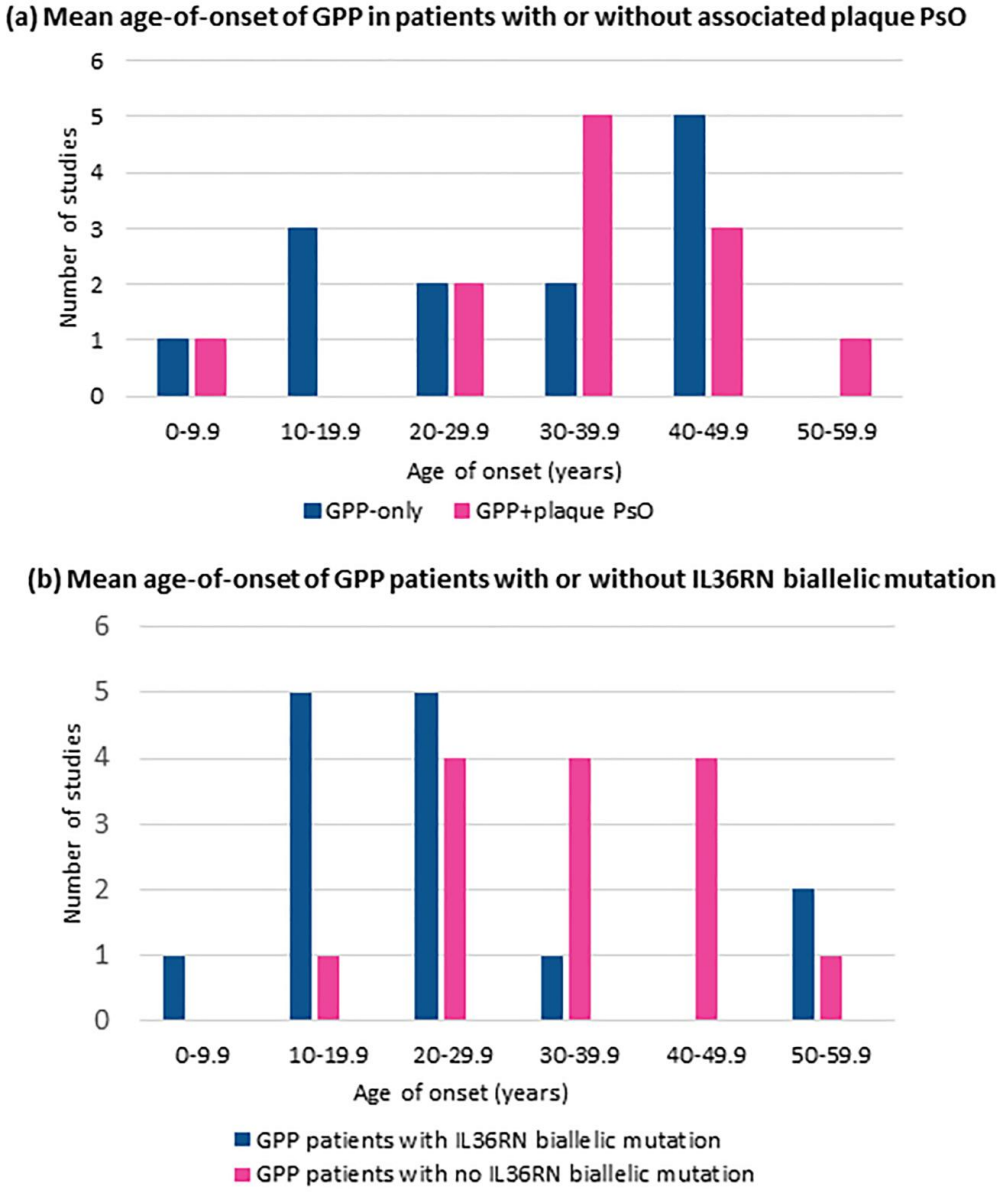
Mean age‐of‐onset in GPP patients by associated plaque PsO and *IL3*6RN variants. (a) Mean age‐of‐onset of GPP in patients with or without associated plaque PsO; (b) Mean age‐of‐onset of GPP patients with or without *IL3*6RN biallelic mutation. GPP, generalised pustular psoriasis; PsO, psoriasis. GPP patients with no *IL36RN* biallelic mutation include patients with either monoallelic or no *IL36RN* mutations.

**FIGURE 6:**
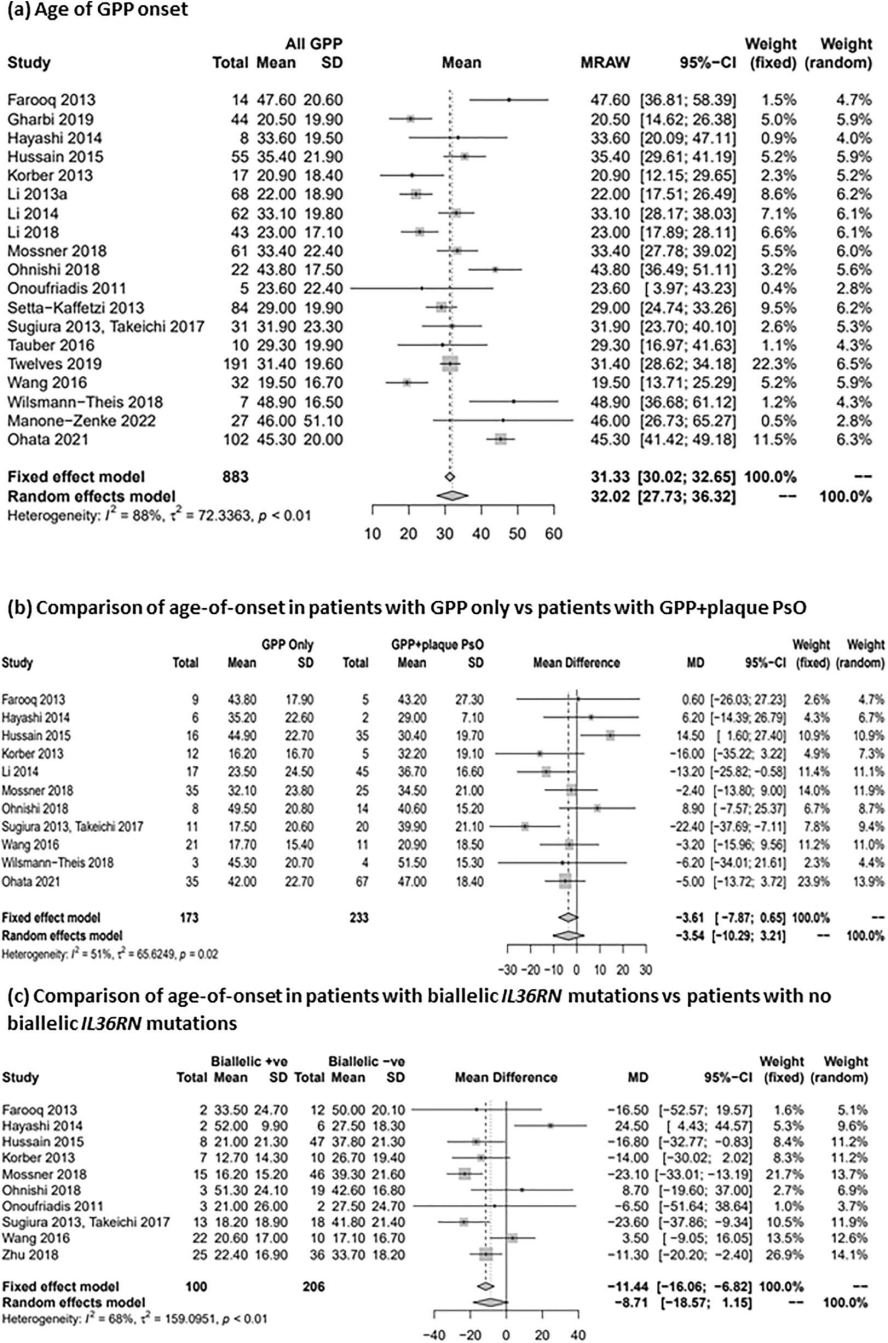
Meta‐analyses assessing age‐of‐onset in patients with GPP. (a) Age of GPP onset; (b) Comparison of age‐of‐onset in patients with GPP only versus patients with GPP + plaque PsO; (c) Comparison of age‐of‐onset in patients with biallelic *IL36RN* mutations versus patients with no biallelic *IL3*6RN mutations. CI, confidence interval; GPP, generalised pustular psoriasis; MD, mean difference; PsO, psoriasis; SD, standard deviation.

GPP age‐of‐onset by mutational status was reported in 22 studies, excluding paediatric studies and one elderly‐onset study. Mean age‐of‐onset ranged from 5.9 to 48.9 years old, with most studies reporting GPP onset at 20–40 years. The mean (95% CI) age‐of‐onset for all patients with GPP was 32.02 years (95% CI 27.73, 36.32; Figure 6a). Meta‐analysis point estimate favoured an earlier GPP age‐of‐onset in the GPP‐only subgroup compared to the GPP + plaque PsO subgroup; however, the results were not statistically significant (mean difference [MD] [95% CI]: −3.54 [−10.29; 3.21]; Figures 5a and 6b). Age of GPP onset was also compared between biallelic *IL3*6RN mutation‐positive and negative patients. The meta‐analysis suggested a younger age‐of‐onset in the biallelic *IL3*6RN mutation‐positive population, by almost 9 years, but results were not statistically significant (MD [95% CI]: −8.71 [−18.57; 1.15]; Figures 5b and 6c).

### 3.4 *HLA‐Cw6* allele frequency

The *HLA‐Cw6* allele was discussed in 6 studies, in which the frequency ranged from 0% to 28.6% (Table S8). A few patients who had the *HLA‐Cw6* allele had a history of plaque PsO, however, history of plaque PsO was inconsistently reported. Arakawa (2018) reported no significant difference in *HLA‐Cw6* allele frequency between GPP patients and the general population.^31^ In one study, 7 of 10 GPP patients had the *HLA‐Cw7* allele instead of *HLA‐Cw6* that is associated with plaque PsO.

### 3.5 Spesolimab and the *IL36RN* mutation

In Effisayil^TM^ 1 of spesolimab, 46 out of 53 patients had genotyping data.^32^ According to a subgroup analysis evaluating the safety and efficacy of spesolimab on the basis of *IL3*6RN mutation status, a high proportion of patients with an *IL3*6RN mutation achieved both the primary and secondary endpoint.^32^ Among patients who had an *IL3*6RN mutation (*n* = 14), complete pustule clearance at week one was achieved by 7 of 8 patients (87.5%) treated with spesolimab versus 1 of 6 patients (16.7%) treated with placebo.^32^ Similarly, among *IL3*6RN mutation‐positive patients, 75.0% who received spesolimab and 16.7% who received placebo experienced clear/almost clear skin (GPPGA total score 0 or 1). In patients without *IL3*6RN mutations, complete pustule clearance and clear/almost clear skin at week one were observed in 42.9% and 28.6% among those treated with spesolimab versus 0.0% and 9.1% of patients who received placebo.^32^

Regardless of mutation status, spesolimab treatment resulted in rapid and sustained pustular clearance and clear or almost clear skin over the course of 12 weeks. Furthermore, the safety profile of spesolimab was similar in patients with and without an *IL3*6RN mutation.^32^

## 4 DISCUSSION

The concept of autoinflammatory keratinisation disease (AiKD) is widely acknowledged in the context of inflammatory keratinisation disorders characterised by genetic autoinflammatory mechanisms.^33^ The recent elucidation of the pathogenesis and pathophysiology of PsO and its related disorders, including its rare subtype, GPP, associated with the hyperactivation of skin innate immunity, suggests they are representative diseases of AiKD.^33^

The distinctions between GPP and plaque PsO are multifaceted; however, differences in genetic aetiology may be most telling and relevant for treatment approach. The first genetic mutation identified in patients with GPP was the homozygous missense mutation c.80 T > C (p.Leu27Pro), which was associated with a deficiency in IL‐36Ra; patients with this mutation exhibited symptoms including high‐grade fever and general malaise in addition to erythema and pustules.^34^ In addition to *IL3*6RN, the mutations/variants detected in genes *CARD14, AP1S3, MPO*, and *SERPINA3* were reported to be genetic causative or predisposing factors for pustular PsO.^33^ In this study, patients with GPP alone presented with *IL3*6RN mutations more frequently than patients with associated plaque PsO, a finding that aligns with previous findings by Zhou et al. (2021),^6^ Liu et al. (2020),^9^ and Choon et al. (2023).^35^ No biallelic mutations were found in plaque PsO‐only cohorts across eight studies that analysed *IL3*6RN mutations in this subgroup; presence of monoallelic mutations ranged from 0% to 11.9%.^19^, ^20^, ^21^, ^22^, ^23^, ^24^, ^25^, ^26^ In clinical practice, a proportion of patients with GPP present with concomitant plaques, but this population has not been well characterised.^5^, ^36^

GPP prevalence and disease characteristics also varied by geography and ethnicity. Chinese studies reported higher mutation rates, as did two small German studies, and some *IL3*6RN mutation types were only present in people of certain ethnicities. Of the ethnicity‐specific mutations, c.115 + 6T > C (p. Arg10ArgfsX1) was the most frequently reported and was predominantly reported in East Asian studies and international studies that included Asian patients, in agreement with the literature. Liu et al. (2020),^9^Zhou et al. (2021),^6^ Trai et al. (2023),^37^ Hsieh et al. (2023),^38^ and Choon et al. (2023)^35^ also reported this mutation as most often present in Asian populations, however our study revealed that it was also the most frequently reported mutation in general. This findings reported in Zheng et al. (2022)^2^ that loss‐of‐function variants of *IL3*6RN were found with greater frequency in patients in China, and in Twelves et al. (2019)^3^ that the greatest prevalence of GPP‐associated *IL3*6RN mutant alleles (28.8%) was found in patients of East Asian descent.

The originally identified mutation, homozygous missense mutation c.80 T > C (p.Leu27Pro), was the fifth most commonly reported mutation in this analysis, exclusive to patients from North Africa. The second and third most commonly reported mutations in this analysis, c.227 C > T (p.Pro76Leu) and c.338 C > T (p.Ser113Leu), are also homozygous missense mutations that impair IL‐36Ra protein expression and inhibit suppression of inflammatory responses.^6^ A recent study reported a novel heterozygous variant c.96 T > G (p.His32Gln) in two cases.^37^

Another distinction between GPP and plaque PsO is the presence of *HLA‐Cw6* alleles, which were previously established as one of the most strongly associated risk alleles for plaque PsO but do not seem to be specifically associated with GPP.^10^ Rates of *HLA‐Cw6* in patients with GPP found in this analysis (0%–28.6%) are similar to reported background rates in the general population (10.5%^11^ to 20%^12^). In contrast, reported rates of *HLA‐Cw6* are 73%^11^ and 100%^12^ in patients with guttate PsO and 35.7%^39^ in patients with plaque PsO. Muto et al. (2014) reported *HLA‐Cw6* as the primary HLA allele associated with plaque PsO (*N* = 200) in the Japanese population.^40^

Ultimately, the clinical manifestations of GPP can be much more severe than other types of psoriasis such as plaque PsO, leading to urgent treatment needs and possibly life‐threatening complications.^1^, ^2^, ^4^, ^5^ These differences between GPP and plaque PsO highlight the need for novel treatment approaches that target the underlying aetiology and provide significant impact on the disease course. Today, patients with GPP are often treated with therapies that do not address the underlying pathology of GPP, do not have robust evidence of safety and efficacy in patients with GPP, and/or may paradoxically induce flares.^1^, ^5^

While advancements in GPP‐specific treatments have been limited,^1^, ^5^ the IL‐36 inhibitor spesolimab was recently approved in the US, the EU, China, and Taiwan for the treatment of GPP flares, and for the improvement of acute symptoms of GPP in Japan.^41^, ^42^ Blockade of IL‐36 receptor signalling with spesolimab was associated with decreases in proinflammatory‐ and neutrophil‐recruitment mediators as well as reduced keratinocyte activation.^43^

Targeted therapy blocking IL‐36 receptor signalling has been demonstrated to be a safe and effective treatment option for GPP,^13^ with a high proportion of patients with an *IL3*6RN mutation demonstrating complete pustule clearance and clear/almost clear skin.^32^ Spesolimab treatment leads to rapid, normalising or downregulating immune cell activation, and inhibiting the inflammatory response, which results in marked clinical improvement.^43^ Given the earlier onset of GPP in those who have biallelic mutations of *IL3*6RN, genetic testing may support streamlining identification and initiation of treatment for patients with a significant predisposition.

### 4.1 Limitations

This review is limited by its targeted nature, small sample sizes of many included studies, and heterogeneity of study designs and objectives. Assessment of *IL3*6RN mutations in plaque PsO‐only patients was limited to studies of GPP that also enroled patients with plaque PsO and therefore may not be representative of that population. Additionally, studies conducted by the same research group likely recruited some patients who were included in their previous studies, potentially breaking the assumption of independent effect sizes, which is required for meta‐analyses. To avoid this issue, only the most recent study from each research group was included in the meta‐analyses. Evidence of heterogeneity was considerable for several analyses (*I* ^2^ between 75% and 100%), especially age‐of‐onset. This may be due to high variability in the patient numbers in included studies and inclusion of several studies with small sample sizes. Moreover, differences in study locations and designs could have introduced heterogeneity into the analyses.

## 5 CONCLUSION

Our findings provide further evidence that GPP is biologically distinct from plaque PsO, as characterised by higher rates of genetic mutations of *IL3*6RN and the lack of association with the *HLA‐Cw6* allele. Reported mutation rates of *IL3*6RN in patients with GPP and the types of mutations varied geographically and in different ethnic groups, respectively. Ultimately, GPP is a serious condition that requires effective and rapid treatment to control symptoms and limit complications of flares. The advent of treatments specific for the IL‐36 pathway offers new, targeted options for effective treatment.

## CONFLICT OF INTEREST STATEMENT

BG is an employee of Boehringer Ingelheim International GmbH. AP and SH are employed by Symmetron Ltd and were commissioned by Boehringer Ingelheim to undertake the targeted literature review and conduct the analyses. The authors report no other conflicts of interest for this manuscript.

## AUTHOR CONTRIBUTIONS

**James G. Krueger**: Conceptualization (equal); data curation (equal); formal analysis (equal); investigation (equal); methodology (equal); writing – original draft (equal); writing – review & editing (equal). **Anna Pagotto**: Conceptualization (equal); data curation (equal); formal analysis (equal); investigation (equal); methodology (equal); writing – original draft (equal); writing – review & editing (equal). **Samuel Haftel**: Conceptualization (equal); data curation (equal); formal analysis (equal); investigation (equal); methodology (equal); writing – original draft (equal); writing – review & editing (equal). **Birgit Gradl**: Conceptualization (equal); data curation (equal); formal analysis (equal); investigation (equal); methodology (equal); writing – original draft (equal); writing – review & editing (equal).

## ETHICS STATEMENT

Not applicable.

## Supporting information

Supporting Information S1

## Data Availability

All data are incorporated into the article and its online supplementary material.
