# Supplementary material for: Targeted review of IL36RN mutations in patients with generalised pustular psoriasis
Source: Skin Health Dis. 2024 Mar 6;4(2):e343. doi: 10.1002/ski2.343 (PMC10988704; doi:10.1002/ski2.343)
Supplement: Supplementary file 1 — Supporting Information S1 [file SKI2-4-e343-s001.docx]

# Supplemental Materials

**Table S1****.** Embase searches (Embase 1974 to 2021 September 27)

| # | Searches | Results |
| --- | --- | --- |
| 1 | (general* adj4 pustular psoriasis).tw. | 1,010 |
| 2 | Zumbusch.tw. | 120 |
| **3** | **1 or 2** | **1,044** |

**Table S2.** Medline searches (MEDLINE(R) and Epub Ahead of Print, In-Process, In-Data-Review & Other Non-Indexed Citations and Daily 1946 to September 27, 2021)

| # | Searches | Results |
| --- | --- | --- |
| 1 | (general* adj4 pustular psoriasis).tw. | 656 |
| 2 | Zumbusch.tw. | 89 |
| **3** | **1 or 2** | **691** |

**Table S3.** The Cochrane Library (September 28, 2021)

| # | Searches | Results |
| --- | --- | --- |
| 1 | generali?ed NEXT4 pustular psoriasis | 129 |
| 2 | Zumbusch | 2 |
| **3** | **1 or 2** | **130** |

**Table S4.** Embase searches (Embase 2021 September 1 to 2022 August 3)

| # | Searches | Results |
| --- | --- | --- |
| 1 | (general* adj4 pustular psoriasis).tw. | 1,106 |
| 2 | Zumbusch.tw. | 124 |
| 3 | 1 or 2 | 1,140 |
| **4** | **limit 3 to dd=20210901-20220803** | **33** |

**Table S5.** Medline searches (MEDLINE(R) and Epub Ahead of Print, In-Process, In-Data-Review & Other Non-Indexed Citations and Daily September 1, 2021 to August 3, 2022)

| # | Searches | Results |
| --- | --- | --- |
| 1 | (general* adj4 pustular psoriasis).tw. | 736 |
| 2 | Zumbusch.tw. | 92 |
| 3 | 1 or 2 | 771 |
| **4** | **limit 3 to dt=20210901-20220803** | **86** |

**Table S6.** The Cochrane Library (September 1, 2021 to August 4, 2022)

| # | Searches | Results |
| --- | --- | --- |
| 1 | generali?ed NEXT4 pustular psoriasis | 148 |
| 2 | Zumbusch | 2 |
| 3 | 1 or 2 | 149 |
| **4** | **Date custom range: 01/09/2021 - 04/08/2022** | **19** |

**Table S7. Publications excluded from full text review**

| **Reference** | **Reason for exclusion** |
| --- | --- |
| Davan-Wetton C, Hassi NK, Ng JC, et al. The analysis of IL-36RA structural dynamics improves pathogenicity predictions for IL36RN variants observed in generalised pustular psoriasis. Pediatric Rheumatology. Conference: 10th Congress of International Society of Systemic Auto Inflammatory Diseases, ISSAID 2019;17. | Outcome out of scope |
| Chabchoub I, Hammami H, Zaouak A, et al. Pustular psoriasis in Tunisia: A 10-year review. Journal of the European Academy of Dermatology and Venereology 2019;33 (Supplement 3):61. | Outcome out of scope |
| Zhang Z, Jin-Hua Xu. Investigation of Psoriasis Susceptibility Loci in Psoriatic Arthritis and a Generalized Pustular Psoriasis Cohort. Journal of Investigative Dermatology Symposium Proceedings 2018;19:S83-S85. | Outcome out of scope |
| Twelves S, Mostafa A, Bata-Csörg Z, et al. Features of pustular psoriasis observed in 863 patients: Clinical and genetic analysis of a multi-ethnic cohort. Journal of Investigative Dermatology 2018;138 (5 Supplement 1):S54. | Outcome out of scope |
| Sugo N, Shiro Y. A family of CADASIL with a background of familial generalized pustular psoriasis. Journal of the Neurological Sciences 2017;381 (Supplement 1):460. | Case study |
| Arakawa A, Vollmer S, Besgen P, et al. Unopposed interleukin (IL)-36 activity promotes clonal CD4+ T-cell responses with IL-17A production in generalized pustular psoriasis. British Journal of Dermatology 2017;177 (5):e280. | Outcome out of scope |
| Arakawa A, Vollmer S, Besgen P, et al. Unopposed IL-36 activity promotes clonal CD4D T-cell responses with IL-17A production in generalized pustular psoriasis. Journal of Investigative Dermatology 2017;137 (10 Supplement 2):S246. | Outcome out of scope |
| Mahil S, Peakman M, Trembath R, et al. Preliminary validation of the interleukin-36 receptor as a potential therapeutic target in psoriasis. British Journal of Dermatology 2017;176 (4):e41-e42. | Outcome out of scope |
| Böhner A, Roenneberg S, Eyerich K, et al. Acute generalized pustular psoriasis treated with the IL-17A antibody secukinumab. JAMA Dermatology 2016;152:482-484. | Case study |
| Arakawa A, Vollmer S, Besgen P, et al. Generalized pustular psoriasis combines signs of autoinflammation and antigen-driven Th17 responses. Journal of Investigative Dermatology 2016;136 (9 Supplement 2):S209. | Outcome out of scope |
| Ebertsch L, Ekici A, Uebe S, et al. Analysis of further genes of the IL-36 pathway in patients with generalized pustular psoriasis from Germany. Medizinische Genetik 2016;28 (1):173-174. | Outcome out of scope |
| Navarini AAS, Simpson MA, Borradori L, et al. Homozygous missense mutation in IL36RN in generalized pustular dermatosiswith intraoral involvement compatible with both AGEP and generalized pustular psoriasis. JAMA Dermatology 2015;151:452-453. | Case study |
| Eskin-Schwartz M, Basel-Vanagaite L, David M, et al. Atzmony, L. Hodak, E. Intra-familial variation in clinical phenotype of CARD14-related psoriasis. Journal of Investigative Dermatology 2015;1):S78. | Outcome out of scope |
| Shu DJ, H. Z. Mutation analysis of IL36RN in patients with generalized pustular psoriasis. [Chinese]. Journal of Clinical Dermatology 2014;43:531-535. | Case study |
| Shu D, Hong-Zhong J. Mutation analysis of IL36RN in a Chinese Daur family with generalized pustular psoriasis. European Journal of Dermatology 2014;24:415-416. | Case study |
| Arostegui JI, Vicente-Villa MA, Chaves A, et al. P02-002-IL36RN mutations in patients with DITRA. Pediatric Rheumatology. Conference: 7th Congress of International Society of Systemic Auto Inflammatory Diseases, ISSAID 2013;11. | Case study |
| Arakawa A, Vollmer S, Besgen P, et al. Interaction between innate and adaptive immune mechanisms in the autoinflammatory pathogenesis of generalized pustular psoriasis. Journal of the European Academy of Dermatology and Venereology 2013;4):50. | Outcome out of scope |
| Marrakchi S, Masmoudi A, Mseddi M, et al. Generalized pustular psoriasis: Clinical and genetic study of 21 Tunisian cases. Journal of the European Academy of Dermatology and Venereology 2013;4):14. | Outcome out of scope |
| Arakawa A, Summer B, Kawakami Y, et al. Link between innate and adaptive immune response in autoinflammatory condition of generalized pustular psoriasis. Journal of Investigative Dermatology 2013;1):S167. | Outcome out of scope |
| Jordan CT, Cao L, Roberson E, et al. Rare and common variants in CARD14, encoding an epidermal regulator of NF-kappaB, in psoriasis. American Journal of Human Genetics 2012;90:796-808. | Population out of scope |
| Pigatto PD, Riva F, Radaelli A. Generalized pustular versus common psoriasis: Differences in the immune response. Acta Dermato-Venereologica 1984;64:20-24. | Outcome out of scope |
| Zachariae H, Overgaard Petersen H, Kissmeyer Nielsen F, et al. HLA antigens in pustular psoriasis. Dermatologica 1977;154:73-77. | Outcome out of scope |
| Verbov J. Generalized pustular psoriasis associated with chronic renal failure. British Journal of Dermatology 1976;94:105. | Case study |
| Karvonen J, Tiilikainen A, Lassus A. HLA antigens in patients with persistent palmoplantar pustulosis and pustular psoriasis. Annals of Clinical Research 1975;7:112-115. | Outcome out of scope |
| Karvonen J. HLA antigens in psoriasis with special reference to the clinical type, age-of-onset, exacerbations after respiratory infections and occurrence of arthritis. Annals of Clinical Research 1975;7:301-311. | Outcome out of scope |
| Sugiura K. [Biomarkers for generalized pustular psoriasis and related diseases]. Nippon Yakurigaku Zasshi - Folia Pharmacologica Japonica 2015;146:252-5. | Study design out of scope |
| Generalized pustular psoriasis. Medical Journal of Australia 1973;1:571-2. | Outcome out of scope |
| Bettley FR, Forman L. Generalized pustular psoriasis. British Medical Journal 1972;1:441-2. | Outcome out of scope |
| McFadyen T, Lyell A. Generalized pustular psoriasis. British Medical Journal 1972;1:630. | Outcome out of scope |
| Baker H. Generalized pustular psoriasis. British Medical Journal 1972;1:506. | Outcome out of scope |
| Baxter DL. Generalized Pustular Psoriasis. Archives of Dermatology 1964;89:877-9. | Case study |
| Euctr D. A study to test BI 655130 in patients with a flare-up of a skin disease called Generalized Pustular Psoriasis. http://www.who.int/trialsearch/Trial2.aspx?TrialID=EUCTR2017-004231-37-DE, 2018. | Outcome out of scope |
| Nct. A Study to Test BI 655130 in Patients With a Flare-up of a Skin Disease Called Generalized Pustular Psoriasis. https://clinicaltrials.gov/show/NCT03782792, 2018. | Outcome out of scope |
| Euctr B. A study to test whether BI 655130 (Spesolimab) prevents flare-ups in patients with Generalized Pustular Psoriasis. http://www.who.int/trialsearch/Trial2.aspx?TrialID=EUCTR2018-003081-14-BE, 2019. | Outcome out of scope |
| Chabchoub I, Hammami H, Zaouak A, et al. Pustular psoriasis in tunisia: A 10-year review. Journal of the Dermatology Nurses' Association. Conference: 24th World Congress of Dermatology. Italy. 2020;12. | Outcome out of scope |
| Haskamp S. Myeloperoxidase modulates inflammation in generalized pustular psoriasis and further rare pustular skin diseases. European Journal of Human Genetics 2020;28 (SUPPL 1):304-305. | Outcome out of scope |
| Sachdeva M, Rankin B, Mufti A, et al. Management of pediatric generalized pustular psoriasis using biologics: An evidence-based review. Pediatric Dermatology 2021;38(SUPPL 1):83-84. | Study design out of scope |
| Awada B, Abdullah L, Kurban M, Abbas O. Comment on 'De novo generalized pustular psoriasis following Oxford-AstraZeneca COVID-19 vaccine': possible role for Type I interferons. Clin Exp Dermatol. 2022 Feb;47(2):443. | Study design out of scope |
| Choon SE, Bachelez H. Time to accurately determine the burden of generalized pustular psoriasis at the population level. Br J Dermatol. 2022 Jun;186(6):919-920. | Outcome out of scope |
| Hayama K, Fujita H, Terui T. Current trend in the treatment of generalized pustular psoriasis in Japan: Results from a questionnaire-based epidemiological study. J Dermatol. 2022 Jul 25. | Outcome out of scope |
| Hüffmeier U, Frey B, Becker I, Atreya I, Berking C, Mößner R, Wilsmann-Theis D, Uebe S, Kirchner P, Haskamp S. 230 Transcriptomes of MPO-Deficient Patients with Generalized Pustular Psoriasis Reveals Expansion of CD4+ Cytotoxic T Cells and an Involvement of the Complement System and Interferon Pathways. J Invest Dermatol. 2021; 141(10):S188 | Outcome out of scope |
| Ito M, Kamata M, Uchida H, Egawa S, Nagata M, Fukaya S, Hayashi K, Fukuyasu A, Tanaka T, Ishikawa T, Tada Y. Ixekizumab rapidly improves inflammatory markers in patients with generalized pustular psoriasis. Br J Dermatol. 2022 Jun 6. | Outcome out of scope |
| Koren J, Mburu S, Trigos D, Damiani G, Naldi L. Generalized pustular psoriasis: the case for rare disease and orphan designation. Br J Dermatol. 2022 Mar 8. | Outcome out of scope |
| Maul J, Kostner L, Ashcroft D, Barker J, Capon F, Griffiths C, Tadashi T, Navarini AT, Iraspen consortium. 087 International Rare and Severe Psoriasis Expert Network (IRASPEN) - A prospective multi-centre pustular psoriasis registry with genotype-phenotype correlation. J Invest Dermatol. 2021; 141(10):S163. | Outcome out of scope |
| Mori T, Yamamoto T. High levels of serum amyloid A in patients with generalized pustular psoriasis: a possible biomarker of generalized pustular psoriasis. Int J Dermatol. 2022 Mar 18. | Outcome out of scope |
| Reich K, Augustin M, Gerdes S, Ghoreschi K, Kokolakis G, Mößner R, Mrowietz U, Navarini AA, Pinter A, Schäkel K, Staubach P, Sticherling M, Thaçi D, Wilsmann-Theis D. Generalized pustular psoriasis: overview of the status quo and results of a panel discussion. J Dtsch Dermatol Ges. 2022 Jun;20(6):753-771. | Study design out of scope |
| Reymundo A, Vilarrasa E, Baniandrés O, Rodríguez-Fernández-Freire L, Feltes R, Llamas-Velasco M, Daudén E. Effectiveness and safety profile of secukinumab for the treatment of patients with generalized pustular psoriasis in daily practice. J Eur Acad Dermatol Venereol. 2022 Jun 11. | Outcome out of scope |
| Ruan SF, Zhang LL, Liu Z, Lin TT, Wang HQ, Xu QY, Xiang N, Cheng B, Liu T, Ha HY, Gong T, Ji C. Real-world data on the clinical use of secukinumab in pediatric generalized pustular psoriasis: A 48-week retrospective study. J Am Acad Dermatol. 2022 May 16:S0190-9622(22)00806-4. | Outcome out of scope |
| Yu N, Qin H, Yu Y, Li Y, Lu J, Shi Y. A Distinct Immature Low-Density Neutrophil Population Characterizes Acute Generalized Pustular Psoriasis. J Invest Dermatol. 2022 Apr 29:S0022-202X(22)00364-5. | Outcome out of scope |

**Table S8. *HLA-Cw6* allele prevalence**

| **Study ID** | **Country** | **Total GPP patients (N)** | **GPP patients with *HLA-Cw6* allele (n)** | **GPP patients with *HLA-Cw6* allele (%)** |
| --- | --- | --- | --- | --- |
| Ozawa 1998 | Japan | 26 | 3^a^ | 11.5 |
| Cardili 2016 | Brazil | 14^b^ | NR | 28.6 |
| Muto 2014 | Japan | 10 | 0 | 0 |
| Arakawa 2014 | Germany | 8 | NR^c^ | NR |
| Arakawa 2018 | International | 8 | 2 | 25 |
| Onoufriadis 2011 | UK | 5 | 0^d^ | 0 |
| Abbreviations: NR, not reported.  ^a^ All patients with the *HLA-Cw6* allele had a history of PsO.  ^b^ This study included generalised forms of psoriasis, including both GPP and erythrodermic.  ^c^ Study unclear; between one and four patients carried the *HLA-Cw6* allele.  ^d^ Patients with the *HLA-Cw6* allele were excluded from the study. | | | | |
